# Caregiver-informed meta-synthesis of caregivers’ experiences with tracheostomy decision-making in pediatrics

**DOI:** 10.3389/fped.2025.1574484

**Published:** 2025-06-09

**Authors:** Daniel Ofosu, Sela Scott, Elise Kammerer, Larissa Lecona, Kristen Gibson, Stephanie Nitschke, Pam Thompson-Kai, Dacia Chiarieri-Hirsch, Nadia Qureshi, Lesley Soril, Michael van Manen, Maria Castro-Codesal

**Affiliations:** ^1^ Department of Pediatrics, Faculty of Medicine & Dentistry, University of Alberta, Edmonton, AB, Canada; ^2^ Department of Psychology, Faculty of Science, University of Alberta, Edmonton, AB, Canada; ^3^ Parent Partner, Department of Pediatrics, Faculty of Medicine & Dentistry, University of Alberta, Edmonton, AB, Canada; ^4^ Medicine Strategic Clinical Network, Alberta Health Services, Edmonton, AB, Canada

**Keywords:** decision-making, tracheostomy, family-caregivers, pediatrics, parent engagement, qualitative meta-synthesis

## Abstract

**Introduction:**

Family-caregivers facing the decision of a tracheostomy for their child must consider many factors beyond the medical procedure itself, such as quality of life, personal values, and overall care goals.

**Objective:**

This study aimed to synthesize the available qualitative literature exploring family-caregivers’ experiences regarding decision-making about tracheostomy for children.

**Methods:**

We conducted a systematic review and meta-synthesis of qualitative studies exploring family-caregivers’ experiences with tracheostomy decision-making for their children. Four parents with lived experience of having to make decisions about tracheostomy for their child were co-investigators for this work. An experienced librarian developed search strategies to systematically search databases for eligible studies. Two researchers independently screened, extracted and conducted thematic analyses of all the included studies. Researchers and parent partners discussed the identified themes, conceptualized them into categories and crafted a coherent narrative that was larger than the individual contributions of each study.

**Results:**

Seventeen studies were included in the meta-synthesis. Our analysis generated seven themes: (1) the initial reaction; (2) what's going to happen to my child; (3) a place for hope; (4) taking on a new role; (5) how are we going to pay the bills; (6) navigating changing relationships; and (7) a new normal: adjusting to life at home. The identified themes indicated that family-caregivers often consider not only the immediate need to extend their child's life during decision-making but also the long-lasting implications for their child's future, their own roles as caregivers, and the impact on their family as a whole.

**Conclusion:**

The qualitative literature provides valuable insights into the multifaceted challenges faced by family-caregivers during decision-making and the critical role of healthcare professionals in supporting families through this journey beyond the initial decision.

## Introduction

With advancements in healthcare and technology, many children survive critical illnesses, resulting in the need for long-term breathing support through tracheostomy (1). Family caregivers of a child being considered for tracheostomy placement often face difficult decisions during their child's critical illness, resulting in high levels of stress and trauma-like experiences (2). Additionally, children who are candidates for a tracheostomy usually have multiple complex care needs, which heighten the pressure on family caregivers’ decision-making processes as they navigate the healthcare system and learn to meet their child's extensive care requirements (3).

Although multiple studies have explored family-caregivers’ experiences regarding decision-making about tracheostomy for their children, a synthesis of this evidence is lacking. This study aimed to systematically synthesize qualitative research to understand pediatric tracheostomy decision-making from the perspective of family-caregivers. The findings will help healthcare professionals (HCPs) grasp current qualitative literature to better understand the various factors that affect family-caregiver decision-making in addition to the immediate medical needs of their child, ultimately improving communication and support for these families.

## Methods

### Study design

We conducted a systematic search of existing qualitative studies on family-caregiver decision-making for pediatric tracheostomy and used meta-synthesis methodology to synthesize key findings from included studies (4, 5). This meta-synthesis is registered in PROSPERO (CRD42021292208).

### Search strategy and data sources

A search strategy (Online Resource 1) was developed for a previous scoping review (2) on the topic in consultation with a research librarian and a multidisciplinary committee. We used free text terms and subject headings for “tracheostomy, “invasive ventilation,” “home or long-term ventilation,” “decision-making,” “patient preference,” and qualitative study,” and searched in Ovid MEDLINE, Ovid EMBASE, EBSCOhost, CINAHL Plus, and Elsevier Scopus (inception to present). We manually searched ProQuest Dissertations, Google Scholar, and the reference lists of included studies to identify additional literature. The search was completed on January 28, 2024.

### Inclusion criteria

Our inclusion criteria were: (1) type of study: qualitative research, and (2) study population and phenomenon of interest: family-caregivers (anyone directly involved in caring for a child aged 0–18 years) with lived experience of pediatric tracheostomy decision-making.

### Data management and extraction

Studies identified by the search strategy were imported into an EndNote library after removing duplicates. Two independent researchers applied the inclusion criteria for title and abstract screening, followed by full-text screening to confirm eligibility. Any disagreements were resolved by a third reviewer. Using a pre-designed standardized Microsoft Excel form, the following data items were extracted: first author, date and country of publication, study aim, patient population characteristics, and key study findings.

### Meta-synthesis

We followed the processes described by Sandelowski et al. and Erwin et al. on synthesising qualitative literature (4, 5). Two researchers independently familiarise themselves with the included studies through repeatedly reading the studies. Using NVivo, they generated codes from the results of the included studies which were relevant to our study. From the initial codes they developed new descriptive themes using inductive thematic analysis (6, 7). The new themes were discussed and agreed upon by the two researchers, and disagreement was resolved through further discussion with a third researcher until consensus was reached. We extracted quotes published in the included studies to illustrate our new themes.

The generated themes were grouped into categories to capture the broader experiences of caregivers from a family-caregiver perspective. Input from parent partners with lived experience was used to contextualize these themes within the categories.

### Parent engagement

Researchers engaged four parent partners from our provincial health authority who have lived experience in deciding on tracheostomy for their child. Three of the parent partners opted for a tracheostomy and one parent decided against tracheostomy for their child. Parent partners were engaged as co-investigators following the “Involve” goal of the i2S Stakeholder Engagement Options Framework (8). Parent partners contributed to the grouping of meta-synthesis themes into categories, interpretation of the study findings from a family-caregiver perspective, and crafting a manuscript with a coherent narrative larger than the individual contributions of each study which is family-caregiver centered (9).

### Quality appraisal

Two researchers independently evaluated the quality of the included studies using the Critical Appraisal Skills Programme (CASP) checklist (10). The CASP validated checklist is used to evaluate appropriateness and clarity of each study related to the study design, methodology, and results. No articles were excluded based on critical assessment.

## Results

**Figure 1:**
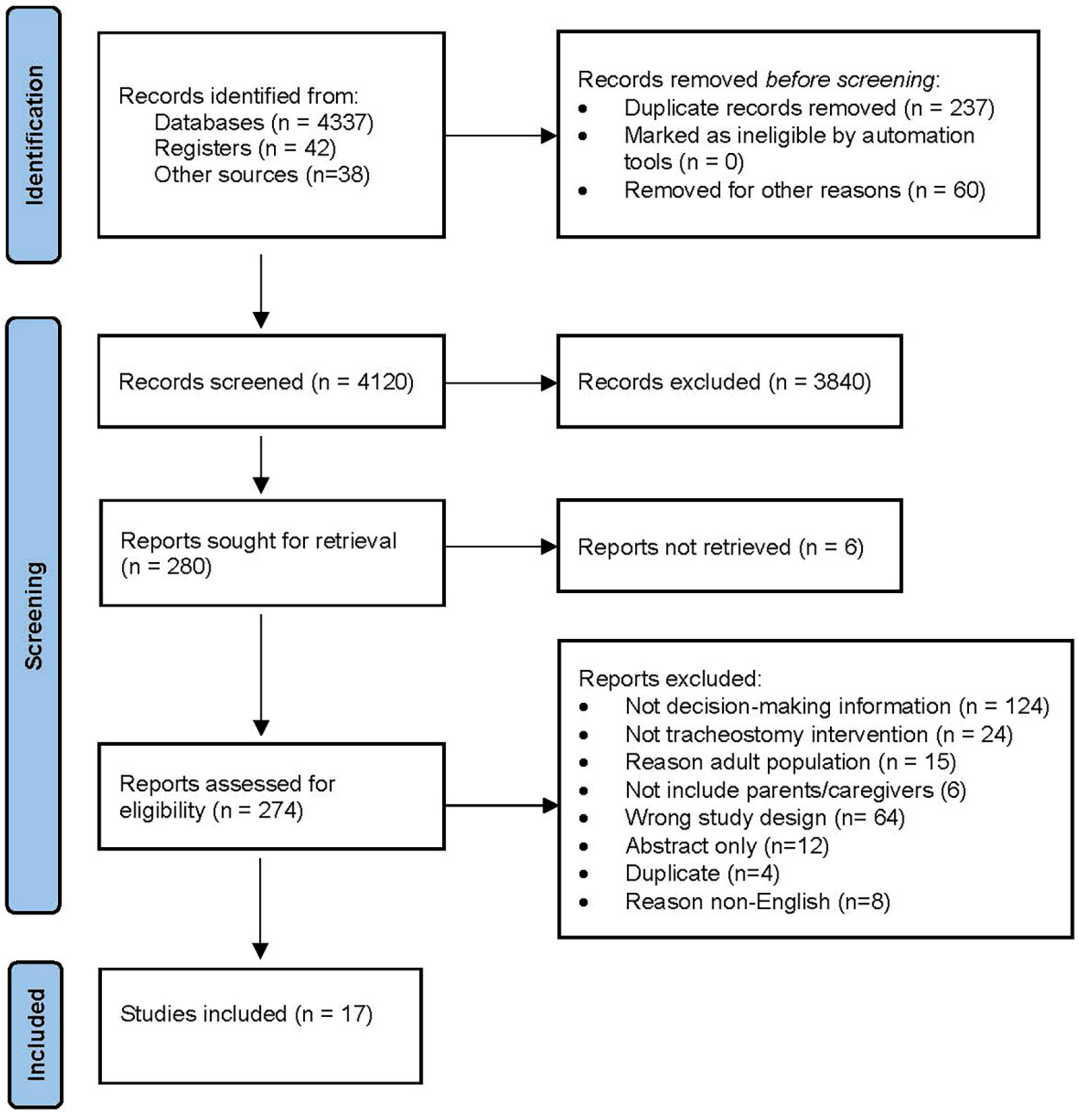
Flow diagram of identification and screening of research studies.

Seventeen studies included in this meta-synthesis (Figure 1) were assessed to meet the inclusion criteria for this meta-synthesis. The quality ratings for each individual study are presented in Online Resource 2. Characteristics of the included studies are summarized in Table 1. Fourteen of the 17 studies were conducted in the United States, with five of them coming from the same dataset (11–15). The sample size of the included studies ranged from 11 to 56. Study participants were predominantly female caregivers (range, 61%–100%). Only one study included caregivers who were facing decisions regarding pediatric tracheostomy (16), four studies included parents who declined tracheostomy, and two studies did not specify.

**Table 1:** Characteristics of included studies.

Reference (year) country	Study aim	Sample size *n* (male/female)	Study participants description	Method of data collection	Data analysis
Acorda et al. (17) (2022) USA	To explore caregivers’ experience with tracheostomy education including barriers and facilitators to learning	23 (8/15)	Parents/legal guardians of 16 children with new tracheostomies	Semi-structured individual interview	Thematic analysis
Bogetz et al. (25) (2022) USA	To retrospectively explore caregiver perspectives on clinician counseling for home mechanical ventilation	26 (4/22)	Parents/legal guardians of 24 children with severe neurological impairment	Semi-structured individual interview	Thematic analysis
Boss et al. (21) (2020) USA	To understand what considerations drive family decisions for, and against, pediatric home ventilation	42 (7/35)	Caregivers of children who faced a decision about home ventilation via tracheostomy	Semi-structured individual interview	Content analysis
Callans et al. (18) (2016) USA	To describe the family experience of caring for a child with a tracheostomy during transition from hospital to home	18 (2/16)	Family members who cared for children who have or had an artificial airway in the home	Semi-structured focus group interview	Content analysis
Carnevale et al. (20) (2007) France/Canada	To examine whether physicians or parents assume responsibility for treatment decisions for critically ill children and how this relates to subsequent parental experience	31 (12/19)	Parents of critically ill children; 9 physicians and 13 nurses who cared for their children	Semi-structured individual interview	Grounded theory analysis
Castro-Codesal et al. (23) (2023) Canada	To gain an understanding of significant aspects of children's tracheostomy	12 (0/12)	Parents whose children are living in the community with a tracheostomy	Semi-structured focus group interview	Grounded theory analysis
Chiang et al. (27) (2021) Canada	To explore the perceptions of caregivers who completed a newly developed long-term ventilation discharge pathway during transition home	11 (3/8)	Family caregivers who have completed a newly developed long-term ventilation discharge pathway as they transitioned home	Semi-structured individual interview	Thematic analysis
Edwards et al. (16) (2020) USA	To assess what families with children with chronic respiratory failure and life-limiting conditions need and want for informed decision-making	44 (10/34)	Parents of 43 children were facing or had previously faced a decision regarding invasive or non-invasive long-term ventilation for their children	Semi-structured individual interview	Thematic analysis
Gower et al. (14) (2020) USA	To identify facilitators and barriers to tracheostomy decision making process	56 (14/42)	Caregivers of 41children with medical complexity who had a tracheostomy	Semi-structured individual/family interview	Content analysis
Henderson et al. (26) (2021) USA	To explore the family experience of home ventilation by comparing expected and real experiences	22 (3/19)	Parents who chose home ventilation for their child	Semi-structured individual/family interview	Content analysis
Jabre et al. (24) (2021) USA	To determine how clinicians can meet the decisional needs of parents considering home ventilation	42 (7/35)	Parents who chose for or against home ventilation for their child	Semi-structured individual/family interview	Thematic analysis
Nageswaran et al. (12) (2018) USA	To assess what families with children with chronic respiratory failure and life-limiting conditions need and want for informed decision-making	56 (14/42)	Caregivers of 41children with medical complexity who had a tracheostomy	Semi-structured individual/family interview	Content analysis
Nageswaran et al. (11) (2020) USA	To explore the role of caregiver religion and spirituality in their decision to pursue tracheostomy for their children	56 (14/42)	Caregivers of 41children with medical complexity who had a tracheostomy	Semi-structured individual/family interview	Content analysis
Nageswaran et al. (13) (2022a) USA	To develop a clinically relevant and realistic model for decision-making about tracheostomy placement	56 (14/42)	Caregivers of 41children with medical complexity who had a tracheostomy	Semi-structured individual/family interview	Content analysis
Nageswaran et al. (15) (2022b) USA	Describe the support needs of caregivers and the resources they use surrounding tracheostomy decision-making for their children	56 (14/42)	Caregivers of 41children with medical complexity who had a tracheostomy	Semi-structured individual/family interview	Content analysis
October et al. (22) (2020) USA	To determine incidence of decisional conflict and regret and contributing factors	39 (14/25)	Parents considering tracheostomy placement for their child	Semi-structured individual interview	Content analysis
Shipman et al. (19) (2023) USA	To describe the rationale of families who decline home ventilation	18 (4/14)	Families who decline home ventilation	Semi-structured individual/family interview	Content analysis

Our analysis of the included studies generated seven themes which were grouped into two broad categories (Table 2).

**Table 2:** Themes and illustrative quotations derived form included studies .

Theme	Contributing studies	Illustrative quotes
Making the decision for tracheostomy		
The initial reaction	Acorda et al. 2022 (17), Boss et al. 2020 (21), Callans et al. 2016 (18), Carnevale et al. 2007 (20), Edwards et al. 2020 (16), Gower et al. 2020 (14), Nageswaran et al. 2018 (12), Nageswaran et al. 2022a (13), October et al. 2020 (22), Shipman et al. 2023 (19)	“I remember whenever they first brought up a trach. I was terrified. I didn't know what to think because I've seen other children with trachs and I just thought it was like the scariest thing possible.” (13) “At first when I tried to talk about it. I said I don't want it because I didn't want a hole in his neck. Then I think it was the best thing for him so he could breathe better and get back to the way he was” (17) “I know my son should not get a trach.. I am making the decision that is best for my son, NOT the decision that is best for me.” (22)
What's going to happen to my child?	Acorda et al. 2022 (17), Bogetz et al. 2022 (25), Boss et al. 2020 (21), Callans et al. 2016 (18), Carnevale et al. 2007 (20), Castro-Codesal et al. 2023 (23), Edwards et al. 2020 (16), Gower et al. 2020 (14), Jabre et al. 2021 (24), Nageswaran et al. 2018 (12), Nageswaran et al. 2022a (13), October et al. 2020 (22)	“We had people that had children with trachs tell us that their child weaned off. The doctors [said] they [didn't] know when because every child is different.” (25) “As it was explained to my husband and I in our “family meeting” with the attending neonatologist the trach was the only option for [our son] to ever be able to leave the hospital. If we chose to not do the surgery we would be “letting him go” and he would not survive without the trach and ventilator.” (22) “I wished someone had said .. “We can put a trach and manage the secretions and help you take her home without having to come back every time she choked or gets a cold and can't breathe. But if the trach does not work, then this is what you're looking at. We will move to hospice.” (25)
A place for hope	Bogetz et al. 2022 (25), Edwards et al. 2020 (16), Gower et al. 2020 (14), Nageswaran et al. 2020 (11), Nageswaran et al. 2022a (13), October et al. 2020 (22), Shipman et al. 2023 (19)	“I thought if I let her die, I was going to hell .. If I wouldn't have gotten her trached, she would have passed away and I felt like I would have gone to hell and I didn't want to go to hell.” (11) “My beliefs played a major role because I have faith in God and I felt confident with pretty much giving the situation to the Lord and feeling okay with it.. Jesus Christ is my savior. That's who I lean on and trust for my guidance and so I just left it in the hands of the Lord and said “Let His will be done.” (13) “[The decision about HMV] was all packaged in the whole “well we'll see how she does” type of a mindset .. There was always that hope that, and especially since they all knew that she had been home without any breathing support for so many months, she could potentially get back to that baseline of not needing support anymore.” (25)
The decisions don't stop there		
Taking on a new role	Acorda et al. 2022 (17), Castro-Codesal et al. 2023 (23), Henderson et al. 2021 (26), Nageswaran et al. 2018 (12), Nageswaran et al. 2022a (15),October et al. 2020 (22), Shipman et al. 2023 (19)	“For me, it was the one thing that I could do – trach care with my eyes closed because it's simple and you have somebody there helping you. But doing a trach change you're actually taking out his airway. It's nerve-wracking.” (17) “Can I do this? Can I do this as his fulltime caregiver? It's a medically fragile infant. Will I be able to handle everything and keep him from getting sick, keep him healthy, keep everything in line?” (26) “It's not the role of caregiver that's frustrating, it is the fragility of the prognosis. I hold my breath all day long waiting for the next “issue’ positive or negative. It's completely unsettling” (22)
How are we going to pay the bills?	Boss et al. 2020 (21), Castro-Codesal et al. 2023 (23), Chiang et al. 2021 (27), Henderson et al. 2021 (26), October et al. 2020 (22)	“… I really wish we had known how much… it's just the financial part. How much loss of income you might there might be.” (26) “The financial concerns are also overwhelming even with the benefits of health insurance” (22) “The financial part. The loss of income. I wish we had known how long it would take for social security to kick in. It's taken a very long time.” (21)
Navigating changing relationships	Boss et al. 2020 (21), Codesal et al. 2023 (23), Edwards et al. 2020 (16), Henderson et al. 2021 (26), Edwards et al. 2020 (16), Shipman et al. 2023 (19)	“It's changed our family dramatically.. my son actually told me that “It feels like you and daddy are divorced because you guys are never together. One goes here and one goes at the hospital.’ We're trying to do what's best for all of our kids it's hard for them as well.” (26) “I reach out to parents—fellow parents that have been at this a while—they are a wealth of information, hundred percent—that's the first place I go to, is other parents” (23) “We had a few comments about how cruel it would be to keep someone alive on the ventilator. It was a disagreement because I wanted her at home, and I saw her still in there, and still interacting with us.” (21)
A new normal: adjusting to life at home	Boss et al. 2020 (21), Codesal et al. 2023 (23), Chiang et al. 2021 (27), Henderson et al. 2021 (26), Jabre et al. 2021 (24)	“[My husband]'s not working… it's hard for us to wrap our brains around him getting a job that would be 9:00 to 5:00 because if I don't have a nurse I can't take my son to school because I can't have both boys in the car by myself.” (26) “No offense to any medical team whatsoever … but you all don't have the home experience. Just being able to talk to the families about something as simple as bath time and going through a grocery store. “How do you do it? Give me tips and pointers.’ Having the family experience is the greatest tool you can have.” (21) “Just walking through that we'll have training and roughly what it will feel like..We will have home nursing, too, which is good and bad. Good is we have the help. Bad is we'll always have someone in the house.” (24)

### Category 1: making the decision for tracheostomy

This category describes the initial reaction of family-caregivers and the immediate need to extend the lives of their children when faced with making the decision for tracheostomy. This category includes three themes: the initial reaction; what's going to happen to my child; and a place for hope.

#### The initial reaction

When physicians first suggest the possibility of a tracheostomy, family-caregivers may experience strong negative emotions such as devastation, fear, worry, overwhelmed and anger (12, 14, 16–19). Family-caregivers must cope with their own emotions while learning a lot of new medical information, not only about tracheostomy but also their child's underlying conditions and how their child's life and their own may change (12).

#### What's going to happen to my child?

Most studies reported that family-caregivers often saw tracheostomy as the only way for their child to go home (12, 14, 16, 20–22). Family-caregivers consistently reported appreciation for honest conversations about goals of care and palliative care approaches, even if they were not their preference. Not having these conversations with HCPs led some family-caregivers, particularly bereaved parents, to feel disappointed about having to raise the option of limiting care and fear being judged as bad parents. During the decision-making process, family-caregivers seek different levels of involvement from HCPs. Some family-caregivers wanted HCPs to be heavily involved in the decision (14, 20, 23) and trusted that physicians were in the best position to advise whether tracheostomy was in their child's best interest (20). Others wanted to weigh the risks and benefits of tracheostomy themselves (20, 24), as they knew their child best (18, 20), considering family values and beliefs (11). The magnitude of their responsibility as decision-makers was not always recognized by HCPs, which negatively affected their relationships with them (20, 22).

Many studies highlighted negative experiences family-caregivers have with HCPs, which impact their decision-making processes and long-term well-being. Factors such as the amount and clarity of information, timing, and setting of tracheostomy discussions significantly influenced their experience. Family-caregivers found depersonalized language, overheard conversations between HCPs in hospital hallways about their child's medical information, and feeling unheard or rushed to be disempowering. Additionally, frequent shift changes and conflicting medical opinions made it difficult to identify who was responsible for their child's care, leading to eroded trust, conflict, and regret (16, 20, 22, 24).

Family-caregivers reported feeling overwhelmed by medical information, yet unprepared for some of the potential long-term risks and challenges associated with tracheostomy, such as potential delays in their child's speech development, ability to eat and drink, and their child's quality of life (13, 16, 21, 23, 25). Other questions that arise early during decision-making include: What support will we have at home? How will we afford it? How will this decision affect our lives, as previously known (16, 24)? Answers to these questions often did not come from HCPs, who often offered an incomplete understanding of life with a tracheostomy, but from other families connected through peer-support programs or social media (14, 16, 21–23).

#### A place for hope

Family-caregivers’ tracheostomy decision-making is not solely based on facts. Some studies have described how family-caregivers’ values, religious beliefs, and spirituality helped them cope with uncertainty and find meaning in their child's illness (13). Some family-caregivers believed that higher power would guide their decision-making and help them find meaning and purpose in the decision to pursue tracheostomy (11, 14, 16). Regardless of the family-caregiver's religion or spirituality, studies have reported that their life values often play a crucial role in the decision-making process, particularly when prioritizing the preservation of their child's life or quality of life over their length of life (13, 14, 19, 25). Conversations with HCPs that allowed room for hope and expression of spirituality were well appreciated, even though HCPs and family-caregivers did not share the same beliefs.

### Category 2: the decisions don’t stop there

This category reflects family caregivers’ decision-making considerations regarding their child's long-term outcomes, the need for new medical skills, shifts in family dynamics, and the lasting impact on home life. These factors influenced their choices early on and continued throughout their child's medical journey. This category includes four themes: taking on a new role, how are we going to pay the bills, navigating changing relationships, and a new normal: adjusting to life at home.

#### Taking on a new role

The literature indicates that becoming a caregiver for a child with tracheostomy is a significant part of the decision-making process for families due to its many implications. Family-caregivers must acquire new skills, including basic care, suctioning, equipment maintenance, and problem-solving when their child becomes ill (12, 17, 19, 22, 26). Many of these routine care skills are complicated, and it takes time for family-caregivers to learn and build the confidence needed to perform them (15, 23). Even after mastering these routine skills, family caregivers often reported significant fears of making mistakes that could harm their child or of being unable to react appropriately in an emergency, potentially leading to hospitalization or even death (12).

#### How are we going to pay the bills?

Studies consistently highlighted that many family-caregivers lived the financial effects of their child's needs early on, even before tracheostomy was decided (22, 26, 27). Most often, family-caregivers had questions about what their healthcare coverage would be for home care and supplies, and whether they would be able to keep going to work at all or whether they would have to reduce hours and assume the financial implications for their family (26). There were also other financial impacts that family-caregivers needed to consider, such as home renovations to improve accessibility for their children or finding funds to pay for the monthly power bill (26).

#### Navigating changing relationships

Family-caregivers acknowledged that the possibility of a tracheostomy brings additional decisions regarding how to navigate existing relationships. While some caregivers believed that their child with a tracheostomy encouraged them and their spouse to bond in caring for their child (26), others faced challenges in their spousal relationships due to time constraints and increased stress levels (26). Family-caregivers acknowledged that their other children also deserved attention, despite the increased unpredictability and limited time in the family's life following tracheostomy (12, 19, 26). These challenges tolled family-caregivers’ health and well-being, as navigating family demands was their main priority. Friendships and community connections also changed during their child's hospitalization, and a sense of isolation was often reported. Family-caregivers, however, recognized that this feeling tend to subside over time and many family-caregivers reported new friendships with other families of medically complex children, either in the hospital or online, with whom they shared experiences, and often received advice to advocate for their child (21).

#### A new normal: adjusting to life at home

Although the included studies focused on tracheostomy decision-making, participants recurrently brought up the moment of returning home after their child had undergone a tracheostomy as a critical step in their child's and family's journey. It also brings more decisions with it. Some family-caregivers reported that many of these new decisions were unexpected. They faced the reality of coping with income loss, managing caregiver turnover, and adjusting to changes in their child's disease trajectory and significant life events, such as starting school (21, 26, 27). Support groups and new friendships with families with similar experiences have become critical support systems for them when facing unexpected decisions (21, 23, 24, 27). Reciprocally, being able to help other families go through similar situations provides family-caregivers with a feeling of satisfaction and an opportunity to offer mutual support and encouragement.

## Discussion

The decision-making process surrounding pediatric tracheostomy is a multifaceted journey fraught with emotional, practical, and ethical considerations for family-caregivers that extends beyond the critical moment of deciding tracheostomy placement. This meta-synthesis is novel at highlighting the challenges associated with family-caregivers navigating uncertainty regarding their child's future. It emphasizes the continuum of family life and the high, sustained level of responsibility required to care for and advocate for a child with medical complexity throughout their life. This study also echoes the real financial burdens that family caregivers face, which are often rooted in systemic societal biases and inequities.

The initial decision about tracheostomy is often a checkpoint for conversations about goals of care, and whether tracheostomy to prolong life is on the child's best interest. Inevitably exacerbated by uncertainty, tracheostomy is often seen by family-caregivers as “the only choice” (12, 14, 16, 20–23). Of concern, some reported insights from family-caregivers may reflect the power imbalance inherent between HCPs and families during the decision-making process. This imbalance can lead to fears of being judged for not fully understanding their child's complex situation or for choosing to go against medical advice. If not addressed, this power imbalance truncates the true shared decision-making process (28) and might contribute to high levels of family-caregiver mental stress. Family-caregivers may feel powerless or undervalued and appoint HCPs as best to decide on their child's care (20, 22). Instead, HCPs should recognize the responsibilities and efforts of family caregivers to make decisions, express genuine interest in their child, and work to establish a trusting relationship. This foundation will allow for safe and respectful conversations at the appropriate time and place (14, 16, 17, 21, 25, 29). These reports are consistent with the growing advocacy work encouraging health systems to incorporate trust and compassion at the core of their institutional values and hired staff accordingly (30–32). Many actions can be taken at the institutional level to emphasize the importance of language, cultivate a caring organizational culture, provide ongoing learning opportunities for HCPs, and co-create resources that enable patients and families to actively participate in decision-making about their loved ones’ health.

Challenges surrounding the initial conversations about tracheostomy have dominated existing qualitative research, which often describes decision-making as a one-time, cross-sectional process with life-changing consequences. From our parent partners, we learned that this emphasis may be misplaced from the family-caregiver perspective. Our parent partners were able to see their experiences reflected in previous qualitative research, particularly the sense of “no choice’ during conversations about tracheostomy. However, they noted that the emphasis in the existing research still tended to focus on the anticipated considerations in the decision-making process, where healthcare professionals are most involved, which may reflect researcher bias. Instead, the initial decision for or against tracheostomy serves as a watershed moment that determines the course of the child's future medical journey and advocacy, requiring ongoing decision-making throughout their life. In fact, the most impactful decisions were not just about tracheostomy placement but also included unforeseen challenges, such as moving their child's room to accommodate necessary equipment, needing to rehome a pet, and advocating for their child's right to attend school. These many other decisions start early in the process, even before tracheostomy is performed, and are present throughout the child's life. Caring for a child with tracheostomy is a long process that require much more effort, endurance, and constant deliberation than the initial decision about whether to pursue the procedure.

Recognizing the complex journey of bringing a child with a tracheostomy home provides valuable insights for HCPs to bridge communication and ideological gaps that can be overlooked during decision-making. Embracing a true family centered approach requires HCPs to support caregivers beyond immediate medical concerns and to address the practical and emotional challenges of an unexpected lifestyle shift. Additionally, peer-mentorship experiences can help future caregivers anticipate the downstream decisions that may follow this critical choice.

A key area for future research is the role of equity-related factors in family-caregiver decision-making in pediatric tracheostomy. Existing studies show that caregivers often assume on medical and managerial roles, hiring and overseeing home care, often at the expense of their own careers (13, 15, 27). These burdens vary depending on societal investments in healthcare, home care programs, and community support. Research shows quantifiable severe burdens on caregivers and yet limited government reimbursements, with many, disproportionally mothers stepping down from careers to become primary caregivers, deepening gender-based inequities (33–36). Additionally, financially vulnerable families may face heightened challenges. Gaps remain in understanding the influence of culture, race, socioeconomic status, and other social determinants of health and their intersections on decision-making, and ultimately, how these affect outcomes for children with tracheostomies (37). Further research is needed to fully explore the impact of these equity-related factors and systemic biases in this context.

Other research gaps include the lack of perspectives from family-caregivers who chose palliative care or experienced child loss, leading to a publication bias favoring those who opted for tracheostomies. Additionally, the experiences of caregivers of children in long-term care, foster medical care, or those involved in conflict resolution or legal actions remain underexplored.

This meta-synthesis is limited by data availability and authors’ interpretation of qualitative data from the included studies, since we interpreted the authors’ interpretations of family voices. Our parent engagement strategies to include parent partners with lived experience might have mitigated this limitation by providing meaning to the study findings and re-focusing on a family-caregiver perspective. Further, parent partners did not identify other aspects integral to family-caregivers’ decision-making processes that were missing from the included studies, but identified a clinician/researcher bias in the interpretation of the data.

## Conclusions

This study provides valuable insights into the decision-making process surrounding pediatric tracheostomy using a family-caregiver centered approach, highlighting the additional challenges faced by family-caregivers during the decision-making process that extend beyond consent for a tracheostomy. HCPs play a critical role in supporting families by fostering open communication, shared decision-making, and empowering family-caregivers to navigate the complexities of pediatric tracheostomy in hospitals and prepare them for returning home.
