# Supplementary material for: Caregiver-informed meta-synthesis of caregivers’ experiences with tracheostomy decision-making in pediatrics
Source: Front Pediatr. 2025 Jun 9;13:1574484. doi: 10.3389/fped.2025.1574484 (PMC12183042; doi:10.3389/fped.2025.1574484)
Supplement: Supplementary file 1 [file Table1.docx]

**Title:** Caregiver-informed meta-synthesis of caregivers’ experiences with tracheostomy decision-making in pediatrics

**Authors:** Daniel Ofosu^1^, Sela Scott^1,2^, Elise Kammerer^1^, Larissa Lecona^3^, Kristen Gibson^3^, Stephanie Nitschke^3^, Pam Thompson-Kai^3^, Dacia Chiarieri-Hirsch^4^, Nadia Qureshi^4^, Lesley Soril^4^, Michael van Manen^1^, Maria Castro-Codesal^1^

1. Department of Pediatrics, Faculty of Medicine & Dentistry, University of Alberta, Edmonton, AB, Canada
2. Department of Psychology, Faculty of Arts, University of Alberta, Edmonton, AB, Canada
3. Parent partner, Department of Pediatrics, Faculty of Medicine & Dentistry, University of Alberta, Edmonton, AB, Canada
4. Alberta Health Services, Edmonton, AB, Canada

**Corresponding author**

Dr. Maria Castro-Codesal

3-518 Edmonton Clinic Health Academy

University of Alberta

11405 87 Avenue NW

Edmonton, AB, T6G 1C9

[castroco@ualberta.ca](mailto:castroco@ualberta.ca)

Search strategy for MEDLINE and adapted for other databases

| 19 Mechanical Ventilation/  20 Tracheostomy/  21 (mechanical adj (respirat* or  ventilat*)).tw,kf.  22 ((long term or longterm) adj5  ventilat*).tw,kf.  23 ((domicil* or home*) adj5  ventilat*).tw,kf.  24 (invasive adj5 ventilat*).tw,kf.  25 invasive respiratory support*.tw,kf.  26 pulmonary ventilat*.tw,kf.  27 (tracheostom* or tracheotom*).tw,kf.  28 ((trachea or nasotracheal or orotracheal  or endotracheal) adj2 (tube* or  intub*)).tw,kf.  29 supraglottic airway*.tw,kf.  30 SGA.tw,kf.  31 Esophageal-tracheal combitube*.tw,kf.  32 Laryngeal Tube*.tw,kf.  33 or/19-32 [INVASIVE VENTILATION]  34 Continuous Positive Airway Pressure/  35 Positive-Pressure Respiration/  36 Ventilators, Negative-Pressure/  37 Hypoventilation/pc, rh, th  38 Interactive Ventilatory Support/  39 Respiration, Artificial/  40 Respiratory Insufficiency/pc, rh, th  41 (continuous positive adj2 (airway* or  air-way* or pressure)).tw,kf.  42 (positive adj2 pressure adj (assist* or  support* or therap*)).tw,kf.  43 ((positive adj2 pressure) & (airway* or  air-way* or breath* or inspirat* or  respirat* or ventilat*)).tw,kf.  44 (continuous negative adj2  pressure).tw,kf.  45 (negative pressure adj2 (respirat* or  ventilat*)).tw,kf.  46 respiratory support*.tw,kf.  47 or/34-46 [GENERAL VENTILATION]  48 invasive*.mp.  49 47 & 48 [INVASIVE VENTILATION]  50 33 or 49 [BOTH INVASIVE  VENTILATION CONCEPTS  COMBINED]  51 exp adolescent/  52 exp child/  53 exp infant/  54 exp minors/  55 exp pediatrics/  56 p?ediatric*.tw,kf. | 57 newborn*.tw,kf.  58 neonat*.tw,kf.  59 prematur*.tw,kf.  60 preterm*.tw,kf.  61 postmatur*.tw,kf.  62 (baby* or babies*).tw,kf.  63 (infant* or infancy).tw,kf.  64 toddler*.tw,kf.  65 (pre?schooler* or pre  schooler*).tw,kf.  66 (school?child* or school  child*).tw,kf.  67 nursery school*.tw,kf.  68 kindergar*.tw,kf.  69 (primary school* or grade  school*).tw,kf.  70 secondary school*.tw,kf.  71 elementary school*.tw,kf.  72 (high?school* or high  school*).tw,kf.  73 child*.tw,kf.  74 (kid or kids).tw,kf.  75 prepubescen*.tw,kf.  76 pubescen*.tw,kf.  77 pubert*.tw,kf.  78 adolescen*.tw,kf.  79 teen*.tw,kf.  80 boy*.tw,kf.  81 girl*.tw,kf.  82 (minor or minors).tw,kf.  83 or/51-82 [CHILD FILTER]  84 50 & 83  85 Clinical decision-making/  86 Clinical Decision Rules/  87 exp Decision making/  88 Decision making, shared/  89 Decision support techniques/  90 Patient preference/  91 Patient participation/  92 ((share* or collaborat* or informed)  adj2 decision*).tw,kf.  93 (decision* adj2 (make* or  making)).tw,kf.  94 (decision* adj2 (support* or  aid*)).tw,kf.  95 (patient* adj2 (prefer* or participat*  or involv* or engag* or  collaborat*)).tw,kf.  96 or/85-95 [DECISION-MAKING]  97 84 & 96 |
| --- | --- |

A validated filter for terms related to pediatric ages has been used. Boluyt N, Tjosvold L, Lefebvre C, Klassen TP, Offringa M. Usefulness of systematic review search strategies in finding child health systematic reviews in MEDLINE. Arch Pediatr Adolesc Med. 2008;162(2):111-6
