# Supplementary material for: Caregiver-informed meta-synthesis of caregivers’ experiences with tracheostomy decision-making in pediatrics
Source: Front Pediatr. 2025 Jun 9;13:1574484. doi: 10.3389/fped.2025.1574484 (PMC12183042; doi:10.3389/fped.2025.1574484)
Supplement: Supplementary file 2 [file Table2.docx]

**Title:** Caregiver-informed meta-synthesis of caregivers’ experiences with tracheostomy decision-making in pediatrics

**Authors:** Daniel Ofosu^1^, Sela Scott^1,2^, Elise Kammerer^1^, Larissa Lecona^3^, Kristen Gibson^3^, Stephanie Nitschke^3^, Pam Thompson-Kai^3^, Dacia Chiarieri-Hirsch^4^, Nadia Qureshi^4^, Lesley Soril^4^, Michael van Manen^1^, Maria Castro-Codesal^1^

1. Department of Pediatrics, Faculty of Medicine & Dentistry, University of Alberta, Edmonton, AB, Canada
2. Department of Psychology, Faculty of Arts, University of Alberta, Edmonton, AB, Canada
3. Parent partner, Department of Pediatrics, Faculty of Medicine & Dentistry, University of Alberta, Edmonton, AB, Canada
4. Alberta Health Services, Edmonton, AB, Canada

**Corresponding author**

Dr. Maria Castro-Codesal

3-518 Edmonton Clinic Health Academy

University of Alberta

11405 87 Avenue NW

Edmonton, AB, T6G 1C9

[castroco@ualberta.ca](mailto:castroco@ualberta.ca)

Quality assessment of the included studies using the Critical Appraisal Skills Program (CASP) Checklist for Qualitative Research

| **Study/Country** | **Section A: Are the results valid?** | | | | | | **Section B: What are the results?** | | | **Section C: Will the results help locally?** |
| --- | --- | --- | --- | --- | --- | --- | --- | --- | --- | --- |
|  | Was there a clear statement of the aims of the research? | Is a qualitative methodology appropriate? | Was the research design appropriate to address the aims of the research? | Was the recruitment strategy appropriate to the aims of the research? | Was the data collected in a way that addressed the research issue? | Has the relationship between researcher and participants been adequately considered? | Have ethical issues been taken into consideration? | Was the data analysis sufficiently rigorous? | Is there a clear statement of findings? | How valuable is the research? |
| Acorda (2022)/ USA | Y | Y | Y | Y | Y | Y | Y | Y | Y | Y |
| Bogetz  (2022)/ USA | Y | Y | Y | Y | Y | CT | Y | Y | Y | Y |
| Boss (2020)/ USA | Y | Y | Y | Y | Y | Y | Y | Y | Y | Y |
| Callans (2016)/ USA | Y | Y | Y | Y | Y | Y | Y | Y | Y | Y |
| Carnevale (2007) / France/Canada | Y | Y | Y | Y | Y | Y | Y | Y | Y | Y |
| Castro-Codesal (2023) / Canada | Y | Y | Y | Y | Y | Y | Y | Y | Y | Y |
| Chiang (2021)/ Canada | Y | Y | Y | Y | Y | Y | Y | Y | Y | Y |
| Edwards (2020)/ USA | Y | Y | Y | Y | Y | Y | Y | Y | Y | Y |
| Gower (2020)/ USA | Y | Y | Y | Y | Y | Y | Y | Y | Y | Y |
| Henderson (2021) USA | Y | Y | Y | Y | Y | Y | Y | Y | Y | Y |
| Jabre (2021)/ USA | Y | Y | Y | Y | Y | Y | Y | Y | Y | Y |
| Nageswaran (2018)/ USA | Y | Y | Y | Y | Y | Y | Y | Y | Y | Y |
| Nageswaran (2020)/ USA | Y | Y | Y | Y | Y | CT | CT | Y | Y | Y |
| Nageswaran (2022a)/ USA | Y | Y | Y | Y | Y | CT | Y | Y | Y | Y |
| Nageswaran (2022b)/ USA | Y | Y | Y | Y | Y | CT | Y | Y | Y | Y |
| October (2020)/ USA | Y | Y | Y | Y | Y | Y | Y | Y | Y | Y |
| Shipman (2023)/ USA | Y | Y | Y | Y | Y | CT | CT | CT | Y | Y |

CT, can’t tell; Y, yes;
